# Look-alike medications in the perioperative setting: scoping review of medication incidents and risk reduction interventions

**DOI:** 10.1007/s11096-023-01629-2

**Published:** 2023-09-09

**Authors:** Alexandra N. Ryan, Kelvin L. Robertson, Beverley D. Glass

**Affiliations:** 1 Pharmacy Department, Townsville University Hospital, 100 Angus Smith Drive, Douglas, QLD 4810 Australia; 2 College of Medicine and Dentistry, James Cook University, Townsville, Australia

**Keywords:** Anaesthetics, Hospitals, Look-alike, Medication safety, Perioperative, Pharmacy

## Abstract

**Background:**

Look-alike medications, where ampoules or vials of intravenous medications look similar, may increase the risk of medication errors in the perioperative setting.

**Aim:**

This scoping review aimed to identify and explore the issues related to look-alike medication incidents in the perioperative setting and the reported risk reduction interventions.

**Method:**

Eight databases were searched including: CINAHL Complete, Embase, OVID Emcare, Pubmed, Scopus, Informit, Cochrane and Prospero and reported using the Preferred Reporting Items for Systematic Reviews and Meta-Analysis Extension for Scoping Reviews (PRISMA-ScR). Key search terms included anaesthesia, adverse drug event, drug error or medication error, look alike sound alike, operating theatres and pharmacy. Title and abstracts were screened independently and findings were extracted using validated tools in collaboration and consensus with co-authors.

**Results:**

A total of 2567 records were identified to 4th July 2022; however only 18 publications met the inclusion criteria. Publication types consisted of case reports, letters to the editor, multimodal quality improvement activities or survey/audits, a controlled simulation study and one randomised clinical trial. Risk reduction intervention themes identified included regulation, procurement, standardisation of storage, labelling, environmental factors, teamwork factors and the safe administration.

**Conclusion:**

This review highlighted challenges with look-alike medications in the perioperative setting and identified interventions for risk reduction. Key interventions did not involve technology-based solutions and further research is required to assess their effectiveness in preventing patient harm.

**Supplementary Information:**

The online version contains supplementary material available at 10.1007/s11096-023-01629-2.

## Impact statements

- Increased identification of common look-alike labelling and packaging issues in the perioperative setting. Increased awareness may encourage proactive risk identification and modification of practices to improve medication handling and patient safety.
- Implementation of practical strategies to improve the safe storage, selection, and administration of high-risk anaesthetic medications, in the high-risk perioperative setting to reduce patient harm.
- Highlights the need for further investigations with robust methodologies to confirm the effectiveness of the risk reduction interventions in clinical practice.

## Introduction

The World Health Organization (WHO) Global Patient Safety initiative states that medication management is an ongoing international concern [1]. Medication errors and unsafe practices are acknowledged as the leading cause of injury and avoidable harm in healthcare systems worldwide [1]. Medication management encompasses many systems and processes involving; manufacturing, procurement, deployment/storage, prescribing, dispensing/supply, administration, and monitoring of medication use. A medication incident or error can be defined as ‘any preventable event that may cause or lead to inappropriate medication use or patient harm, while the medication is in control of the healthcare professional, patient or consumer’ [2]. With the number of steps involved in these systems and processes, not to mention the inclusion of stakeholders and professional groups, a medication incident is somewhat predictable. Moreover, complexity within medication management may escalate further in the context of an already complex hospital environment, potentially leading to patient harm [3].

Medications are the most common treatment used within the healthcare environment and specifically within the perioperative environment. Boytim et al. published a systematic review looking at the factors contributing to perioperative medication errors and demonstrated a variability in reporting of the incidence of medication errors between different publications [4]. Nanji et al. reported that medication errors occur in 1 out of 20 medication administrations with nearly one third resulting in patient harm [5]. However, Webster et al. used voluntary reporting of errors in the perioperative setting and reported that one drug administration error was reported for every 133 anaesthetics [6], while Mellin-Olsen et al. estimated that anaesthetic-related deaths occur infrequently at a reported rate of less than 1 in 100,000 patients [7].

Medication labelling and packaging, as well as drug naming are critical considerations for safe medication management in clinical practice and similarities may result in Look-alike Sound-alike (LASA) medications. Long standing concerns have been reported with inconsistencies and inadequacies in medication labelling [3]. LASA medications are important in a perioperative setting as anaesthetists administer multiple medications during an operation, with these medications being mostly intravenous, have varying modes of action and often with a narrow therapeutic index. Fonts, shade, and size of ampoules are limited and may be similar for medications produced by the same manufacturer and between manufacturers, resulting in Look-Alike (LA) medications. Syringe labelling can produce a similar LA presentation of medications, however only manufacturer-produced ampoules or vials with ‘original’ labelling or packaging were included in this scoping review. Poor lighting, interruptions, emergency situations, fatigue and stress remain ongoing pressures facing the safe selection and administration of these medications [8]. Sound-alike medication issues, where medication names sound similar, are an important source of error, however contributing factors and risk mitigation interventions associated with these are not included within this scoping review.

To improve medication safety, the interventional approach needs to be multifaceted, as human factors and lack of organisational structure contribute in up to 87% of medication incidents [9–11]. The perioperative care setting is a high-risk area, where many medications are prescribed and administered by anaesthetists, who are specialist physicians involved in the care of patients before, during and after surgery. The practice of anaesthetics is generally autonomous, which consequently places a responsibility back onto the anaesthetists to develop safe practices regarding checking of medications [12]. Unequivocally, reading the medication label prior to administration is a primary measure for ensuring medication safety with intravenous medications. However, a Canadian study found that only 47.6% of practitioners read the label and most likely what determined the selection of the correct medication was the colour of the label [13]. Further, it has been determined that anaesthetists considered label colouring to be an important factor when identifying a medication [13]. Reliance on colour for the safe selection of medications is fundamentally flawed and an unsafe practice as it is well known that people tend to see what they expect to see [14]. Colour can be a prompt or supplemental to checking a medication, however the primary mode of checking should always be the careful reading of every label and this may not be occurring consistently [3].

The Australian Commission for Safety and Quality in Healthcare (ACSQHS) provides guidance around the principles for the safe storage and selection of medications [15]. These strategies may also be deployed at the bedside, whilst an anaesthetist is providing care directly to patients. These guidelines also outline opportunities to improve safety within storage systems in the perioperative and pharmacy environments (refer to Supplementary Information: Table 1) [15]. Overall, there is evidence pertaining to medication errors, LA errors and risk reduction interventions within the published literature. However, there are no focused reviews of LA medication errors and risk reduction interventions in the perioperative setting.

**Table 1:** Inclusion and exclusion criteria for the scoping review based on the participants, concept, context (PCC) framework

	Included	Excluded
Participants	Anaesthetics Anaesthetist Nurses Pharmacists Pharmacy	
Concept	Look alike sound alike (LASA) medications Medication safety risk reduction strategies Drug storage Drug labelling and labelling design Human factors Drug shortage Simulations Sterile cockpit Workspace design and layout Staff perceptions Culture and incivility Smart phones Technology Anaesthetic errors/incidents LASA errors/incidents Staff perceptions Culture	Drug naming Syringe labelling Drug stability Surgical fires Surgical lasers Surgical drapes Surgical instruments Wrong site surgery Retained objects Surgical infections & infection control Occupational exposure Pharmacogenetics Anaesthetic gases ICU/PACU clinical handover Blood management including exsanguinators/tourniquets Surgical checklists Surgical counts Scheduling & cancellations Medication safety Incidents/Errors outside the anaesthetics environment Non-English
Context	Operating theatres Post anaesthetic care unit Inpatients within the hospital environment	Other care environments such as ambulatory care

## Aim

The aim of this scoping review was to explore the published literature related to LA medication incidents, due to the labelling and packaging of intravenous medications, in the perioperative setting and identify reported risk reduction interventions.

## Method

This scoping review followed both the Arksey and O’Malley framework [16] and the Joanna Briggs Institute (JBI) guidelines for scoping reviews [17]. The Preferred Reporting Items for Systematic Reviews and Meta-Analysis extension for Scoping Reviews (PRISMA-ScR) [18] were also utilised to report this review.

### Inclusion criteria

Studies conducted in any healthcare facility (e.g., tertiary referral or other) or clinical setting (e.g., operating theatres or ambulatory care) with no restriction on region, country or geographical area were considered for this review. There were no restrictions placed on study design or study setting (e.g., hospital or laboratory based) however the origin of the label was restricted to the original packaging produced by the manufacturer, rather than labelling of prepared syringes or infusion bags. The search included studies published in English, which included primary research articles, case reports, editorials and newsletter articles. The reason for the exclusion is categorised according to the Participants, Concept, Context (PCC) (Table 1).

### Search strategy

Studies to be included in this review were identified using electronic searching of the CINAHL Complete, Embase, OVID Emcare, Pubmed, Scopus, Informit, Cochrane and Prospero databases from the earliest records of 1952 to 4^th^ July 2022**.** Key search, MeSH headings and synonyms included “anaesthesia”, "adverse drug event" or "drug error* or "medication error*", “look alike sound alike”, “operating theatres” and “pharmacy”. Terms were searched in the databases and a combination of search terms used (refer to Supplementary Information SI: Table 2). In addition, references lists in the articles were screened to identify potential articles missed by the electronic search. The identified articles were analysed, and any further appropriate articles based on title and abstract were also retrieved.

A first review of all relevant titles and abstracts was conducted independently to remove any articles that did not meet the inclusion criteria (Table 1). The full text articles were assessed by all authors and disagreements were resolved through consensus. The data charting process was also conducted independently by the first author and the outcomes were reviewed and approved by all authors.

### Data extraction

The EndNote Library was utilised as a data management tool for the search results to allow collation and screening of search results. Data extraction of all full text articles was performed using a standardised data extraction table. Two authors independently evaluated the full reports for eligibility. Data were extracted by reading the full text articles and collating most relevant fields using Microsoft Excel®, in a format as outlined in the JBI guidelines [17]. Data extracted from the publications included author, year, study location, publication type, study population, aims of the study, methodology, outcome measures and important results.

### Synthesis

A narrative approach to data synthesis was employed to collect the evidence surrounding LA in the perioperative setting and identify alignment to key risk reduction interventions published within the literature. Data were analysed descriptively due to the variability in study methods and nature of the publication. Some of the variables (e.g., year of publication, publication country) were categorised into groups. Countries were categorised as high-income and low- and middle-income based on 2021–2022 World Bank Classification [19].

## Results

**Fig. 1:**
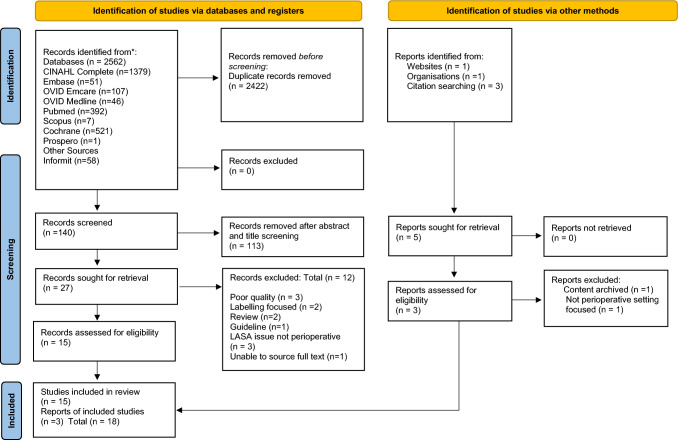
Scoping review PRISMA [18] flow diagram describing the records found and evaluated

As shown in the PRISMA extension for scoping review [18] flow diagram (Fig. 1), the search resulted in a total of 2567 studies identified and screened for inclusion, with 18 publications (0.06% of identified and screened studies) describing LA issues in the perioperative setting included in the final review.

### Study characteristics

Table 2 summarises the published literature related to LA medication incidents and interventions associated with labelling and packaging in the perioperative setting. Of the 18 included publications, 44.4% (n = 8) were published in the last six years. Methodologically, 55.6% (n = 10) were a Letter to the Editor or Case Report. The country of publication origin was most frequently from the United Kingdom (22.2%, n = 4), United States (16.7%, n = 3), and Australia (16.7%, n = 3). According to The World Bank income classifications, 77.8% (n = 14) of the publications were from a high-income country and 22.2% (n = 4) were from either a low- or middle-income countries. All publications originated from a hospital-based setting and anaesthetists were the target for all publications, however 55.6% (n = 10) also had a multidisciplinary focus. Each publication was awarded a points-based classification based on the rigor of the literature using the same approach as Wahr et al. [20]. The results showed that only 27.8% (n = 5) of the publications received a score of 6 or above and 72.2% (n = 13) received a score of 4 or below. ‘Wrong drug’ medication errors were reported in 94% (n = 17) of publications. Patient harm was reported in 22.2% (n = 4) of the publications, with the remainder stating an awareness of the risk that existed.

**Table 2:** Studies that reported on look-alike (LA) medication errors and interventions with labelling/packaging in the perioperative setting

Author, Publication year, Country	Aims of the study	Study design/setting/population	Points awarded	Medication error type	Patient harm reported	Recommendations	Extent of implementation	ACSQHC principles of the safe storage and selections of medications applied to the perioperative setting	Technology based solution?
Estock et al. 2018 [21] United States	To quantify the impact of label design on a realistic, high stress clinical situation	Controlled Simulation Study Hospital Anesthetists and Nursing Trainees	8	Wrong Drug	N	Team - Simulation exercises Labelling - Label design	S	Positive performance shaping factors Education and competency validation Situational awareness and critical thinking Differentiate items	N
Wang et al. 2017 [22] China	To clinically evaluate a type of patented automated anesthesia cart in medication administrations in anaesthesia	Randomised (open label) Clinical Trial Hospital Anaesthetists/ Pharmacists	8	Wrong Drug, Dose Omission, Other	N	Storage - Computerized anesthesia carts Standardisation - Organise and Standardise medication drawers	I	Positive performance shaping factors Education Situational Awareness and Critical Thinking Constraints, barriers and forcing functions Add redundancy	Y
Graudins et al. 2016 [23] Australia	Document the Root Cause Analysis (RCA) interventions surrounding the safe selection and administration of Neuromuscular Blocking Agents (NMBs)	Multimodal Quality Improvement Activity-audit with an intervention Hospital Multidisciplinary (Medicine, Nursing and Pharmacy Staff)	6	Wrong drug	Y	Regulation Industry Procurement - Risk assessment Standardisation - Guideline Labelling - Inhouse Pharmacy labelling Administration - Double checking	I	Risk assessment Positive Performance shaping factors Education and competency validation Limit access or use Constraints, barriers and forcing functions Differentiate items Add redundancy Use of affordances Standardisation	N
Neily et al. 2018 [24] United States	Primary aim was to provide in depth information on reported anesthesia-related adverse events, with root causes and suggested areas for improvement Secondary aim was to recommend actions human factors engineering (HFE) principles to prevent future occurrences of similar events	Retrospective Audit Hospital Multidisciplinary (Medicine/Nursing/Pharmacy)	6	Wrong Drug, Wrong Conc., Improper Dose, Other	Y	Procurement - Prefilled syringes Standardisation Labelling - Bar code medications for scanning Team - Communication Storage - Avoid LA combinations Administration - SMART pumps	R	Positive performance shaping factors Education Limit access or use Constraints, barriers & forcing functions Differentiate items Add redundancy Standardisation	Y
Shultz et al. 2010 [25] Canada	To identify the anesthetic medication to include and to determine how they should be grouped and positioned in a standardised anesthesia medication cart drawer	Multimodal Quality Improvement Activity-survey, observational Hospital Multidisciplinary (Medicine/Anesthetic Assistants /Pharmacy)	6	Wrong Drug, Wrong Conc	N	Standardisation - Organise and Standardise medication drawers Storage - Avoid LA combinations - Visually differentiating hazardous medications	I	Positive performance shaping factors Differentiate items Standardisation Simplify	N
Arnoldus Neetens et al., 2019 [26] The Netherlands	Investigate whether guidelines regarding medicines that should be present on anesthesia carts exist Create an overview of medicines present on anesthesia carts in operating rooms in the Netherlands	Letter to the Editor, including a survey Hospital Anaesthetists	4	Wrong Drug	N	Standardisation - Organise and Standardise medication drawers	R	Positive Performance Shaping Factors Standardisation	N
Imran et al. 2009 [27] Pakistan	To check compliance of anesthetist to current policies set for the use of medication within the operating room and for the induction room floor stock	Observational Audit Hospital Anaesthetists	4	Wrong Drug, Wrong Conc	N	Standardisation - Organise and Standardise Medication Drawers - Adherence to procedures Team - Education	I	Education Standardisation	N
Smellie et al. 1982 [28] Scotland	To discover how nurses and anesthetists locate a drug container in order to read its label and verify its contents	Interviews Hospital Anaesthetists/Nurses	4	Wrong Drug	N	Industry - Colour coding Standardisation - Organise and Standardise medication drawers	R	Differentiate items Use of affordances	N
Zoppellari et al. 2007 [29] Italy	To document three cases of misidentification of propofol drug packaging and the risk reduction strategy deployed	Case Report Hospital Multidisciplinary (Medicine, Nursing and Pharmacy Staff)	4	Wrong Conc	Y	Standardisation - Reporting incidents Storage - Avoid LA combinations Administration - Double checking	I	Positive performance shaping factors Limit access or use Simplify	N
Arora et al. 2011 [30] India	To document a case report of a near miss involving ondansetron and tramadol due to similar coloured packaging	Letter to the Editor Hospital Anaesthetists	2	Wrong Drug	N	Regulation Industry Environmental	R	Positive performance shaping factors	N
Astin et al. 2015 [31] United Kingdom	To document a case report of a near miss involving levobupivacaine and saline 0.9% due to similar coloured packaging	Letter to the Editor Hospital Anaesthetists	2	Wrong Drug	N	Regulation Industry Procurement	R	Education Differentiate items	N
Cohen et al. 2015 [32] United States	To document a case report of a mix up between prochlorperazine and phenylepherine after incorrect removal from an Automated Dispensing Cabinet (ADC) and subsequent administration to a patient	Case Report Hospital Multidisciplinary (Medicine, Nursing and Pharmacy Staff)	2	Wrong Drug	Y	Storage - Optimising the use of Automated Dispensing Cabinets (ADC’s)- barcode scanning, profiling and a physical barrier - Separation of LA	I	Positive Performance shaping factors Limit access or use Constraints, barriers & forcing functions Add redundancy	Y
Dsouza et al. 2017 [8] India	To express opinion related to medication safety in the operating room where multifactorial sources of error are discussed	Letter to the Editor Hospital Multidisciplinary (Medicine, Nursing and Pharmacy Staff)	2	Wrong Drug	N	Regulation Procurement - Risk assessment Standardisation - Organise and Standardise Medication Drawers - Incident analysis Team - Communication Storage - Labelling shelves - Separating LA - Wrapping of medications to distinguish LA Labelling - Double checking- read aloud Administration - Bar code scanning at the point of care	I	Risk assessment Positive Performance shaping factors Education and competency validation Limit access or use Constraints, barriers and forcing functions Differentiate items Add redundancy Standardisation	Y
Goresky et al. 1994 [33] Canada	To document a case report of lookalike packaging and to raise awareness	Letter to the Editor Hospital Anesthetists/Pharmacists	2	Wrong Drug	N	Procurement Standardisation - Risk assessment Team - Communication Storage - Avoid LA	I	Risk assessment Positive performance shaping factors Limit access or use Constraints, barriers and forcing functions	Y
Leng et al 2002 [34] United Kingdom	To express an opinion related to the multifactorial causes of medication error in the anesthetic room that are not limited to drug labelling	Letter to the Editor Hospital Anesthesiologists	2	Wrong Drug	N	Procurement - Consultation/ communications Environmental - Limit stock in the workspace Standardisation - Organise and Standardise medication drawers Storage - Segregate bulk stock Administration - Double checking	R	Positive performance shaping factors Education Limit access Add redundancy	N
Marshall et al. 2019 [35] Australia	To examine the causes of medication handling problem and discuss solutions to address the human factors considerations	Editorial Hospital Anaesthetists	2	Wrong Drug	N	Industry Procurement	R	Positive performance shaping factors Education Constraints, barriers & forcing functions	N
Watts et al. 2016 [36] England	To document concerns related to lack of a standardised approach to drug packaging and the risk to patient safety	Letter to the Editor Hospital Anaesthetists	2	Wrong Drug	N	Regulation Industry Environmental	R	Positive performance shaping factors	N
Wong 2015 [37] Australia	To express opinion related to medication safety in the operating room and state that improved labelling was demonstrated with the Codonics Safe Label System	Letter to the Editor Hospital Anaesthetists	2	Wrong Drug	N	Environmental Team - Role definition Labelling - Improved labelling via technology	Nil	Positive performance shaping factors Education Add redundancy	Y

**Key: Points Awarded:** Based on the quality of the literature sources and an explanation is provided within the method section

**Medication Error Type:** Based on the National Coordinating Council for Medication Error Reporting and Prevention (NCC MERP), NCC MERP Taxonomy of Medication Error [38]. Drug Concentration abbreviated to Drug Conc

**Patient harm** determined if specially mentioned as occurring due to LA issue in the publication

**Theme Classification**: Publications were awarded a points based allocation based on the rigor of the literature and was based on the approach used by Wahr et al. [20]: Explanation of the point allocation; 8: Recommendations based on a scientific design, 6: Recommendation based on formal consensus of experts or rigorous review of the literature, 4: Recommendation by a group of experts (not reaching a formal consensus or guideline level, but where the experts are widely published in the field of medication safety), 4: Recommendations based on a case series, such as surveys to collect recollections of errors, 2: Individual case reports and editorials by a single individual

**ACSQHC Principles of the safe storage and selections of medications **[15]**:** Proposed interventions categorised according to the 14 principles for application within pharmacy and clinical areas: 1. Risk Assessment, 2. Checklists and reminders, 3. Positive performance shaping, 4. Education and competency validation, 5. Situational awareness and critical thinking, 6. Recovery and harm mitigation, 7. Limit access or use, 8. Constraints, barriers and forcing functions, 9. Differentiate items, 10. Add redundancy, 11. Optimise display of medicines information, 12. Use of affordances, 13. Standardise, 14. Simplify

**Extent of Implementation:** Categorised accordingly to the extent intervention(s) being implemented into clinical practice: I: Implemented, R: Recommended, S: Simulation, Nil: Nil Intervention (opinion only)

**Technology Based Solution:** The author determined if the proposed intervention is based on computerised technology that may have cost implications

Interventions to reduce the risk of LA medications were reported to have been recommended in 44% (n = 8) of publications and the implementation of risk mitigated strategies had actually occurred in 44% (n = 8) of publications (Table 2). There were nine themes and 27 subthemes identified relating to LA medication incidents in the perioperative setting (Table 3). The mean number of interventions or risk reduction themes identified from each publication was 4.00 (SD = 1.60). Common themes identified: organising and standardising medication drawers; avoiding LA combinations together; risk assessments; team communication for procurement; improved regulation and industry responsibility and education with clinicians to raise awareness of the risks (Table 3).

**Table 3:** Summary of the risk reduction themes and intervention subthemes in the published literature related to medication management and LA issues in the perioperative setting

Intervention theme	Intervention sub theme
Regulation	Manufacturer labelling and packaging e.g. colour coding
Procurement	Risk assessment
	Communication and consultation
	Prefilled syringes
Standardisation	Standardising and organising medication drawers
	Guidelines
	Adherence to procedures
	Incident analysis
Labelling	Barcode medications for scanning
	Improved labelling via technology
	Label design
	Inhouse pharmacy labelling
Environmental	Limit medications in the workspace
Team	Communication
	Education
	Role definition
	Simulation exercises
Storage	Avoid LA combinations
	Computerised anaesthesia carts
	Optimise use of the automated dispensing cabinets (ADCs)
	Label shelving
	Visually differentiate medications
	Segregate bulk stock
Administration	Double checking
	Double check with a read aloud
	Barcode scanning at the point of care
	Smart pumps

The mean number of ACSQHS guidance principles identified per publication was 3.78 (SD = 3.70). Commonly identified themes included positive performance shaping factors; standardisation, constraints, barriers and forcing functions; limiting access; differentiating items; adding a redundancy (double check) and education to raise awareness of the risks (Table 2). A technology-based solution was determined by the authors to be those that required a computer-based solution that is likely to have cost implications. Technology based solutions were however identified or recommended in only 27.8% (n = 5) of publications with the remaining 72.2% (n = 13) of risk reduction strategies involving low technology interventions (Table 2).

## Discussion

To our knowledge, this is the first scoping review to identify LA medication incidents in the perioperative setting and report on risk reduction interventions. The most frequently reported incident type for LA medications involves the ‘wrong drug’, where an incorrect medication is selected for administration. Risk reduction interventions (themes and subthemes) in (Table 3) are aligned to aspects of the medication management cycle ranging from regulation and through to administration. Interventions were often found to be independent of technology and therefore cost of interventions is unlikely to be a barrier. The best practice guidelines ‘Principles for the safe selection and storage of medicines’ produced by Australian Commission on Safety and Quality in Healthcare provide recommendations for the management of medications, particularly LA medications [15]. This scoping review also assesses the alignment of these principles to intervention themes in the literature (Table 2).

A limitation was that studies were restricted to those published in the English language, due to lack of translation resources.

This review adds to the existing knowledge of LA in the perioperative clinical setting. Regulation and procurement of these medications present opportunities for interventions that contribute to medication safety before a medication even enters the hospital setting. The importance of appropriate regulation governing the labelling and packaging of medications and the role of pharmaceutical industry in completing the appropriate premarket research into labelling and packaging review was highlighted [8, 23, 30, 31, 36]. Manufacturer colour coding of labelling and packaging has been proposed as one potential intervention to better identify similar medications and prevent unintended LA ampoules and vials before they reach the hospital environment. In Australia, a project [23] completed multiple interventions in response to Root Cause Analysis of neuromuscular blocking medications. This resulted in the Therapeutic Goods Administration (TGA) mandating red banding and wording on all medications within this class to avoid LA medication errors. There are no consistent approaches to the colour coding, container size, background or font size between countries, consistent premarketing surveillance approaches focusing on labelling for legibility, ease of identification and avoidance of LA labels [3]. Differentiation is a key ACSQHC principle for the safe storage and selection of medication [15] and this was identified throughout the published literature [8, 23–25, 31].

The Institute for Safe Medication Practices (ISMP) National Medication Errors Reporting Program (ISMP MERP) [39] published a summary of common factors associated with labelling and packaging issues based on voluntary reporting associated with clinical incidents (refer to Supplementary Information: Table 3). This table provides a useful summary to refer to in clinical practice when conducting clinical incident reviews or near miss investigations and the authors would recommend the inclusion into local risk assessment processes.

Interdisciplinary collaboration between anaesthetists and pharmacists for procurement decisions impacting the perioperative setting has been proposed as means of identifying LA ampoules or vial combinations before reaching a patient [3, 33, 34]. Medications should be risk assessed to avoid LA combinations collaboratively and consideration given to alternative presentations of the medication, such as prefilled or ready to use syringes, with appropriate labelling where required [3]. Pre-filled syringes reduce the number of steps in transferring the medication from the ampoule prior to administration, produced by the local pharmacy department or external supplier and are desirable for convenience however may be cost prohibitive for some organisations [3]. Pre-filled syringes for targeted medications, such as high risk medications, including neuromuscular blockers should be considered for implementation into clinical practice.

Standardisation and organising of medication storage was shown to be a fundamental safety strategy. A prospective open label clinical trial assessing the clinical impact of automated versus manual anaesthesia drawers showed a statistically significant reduction in incidents with medication documentation involving the use of automated drawers [22]. Shultz et al. [25] considered the standardisation of conventional manual drawers by: separation of similar looking medications by having a standardised list with medication groupings and positioning according to order of use, similarity of action and also risk of misuse. Similarly, a study by Arnoldus Neetens et al. [26] focused on standardising anaesthesia drawers. Practice supported by guidelines and adherence to procedures were also found to be an important intervention for standardisation [15] which limited variability and the potential for incidents [24]. Incident review processes incorporated in Anaesthetic and Pharmacy Departments that provides dialogue and feedback for medication incidents and near misses, where a focus on learning and preventing future incidents was found to be important [3, 8]. Open discussion of medication incidents is recommended, in particular examples of where positive learnings were identified.

The ACSQHC Principles for the safe storage and selection of medication [15] defines ‘Positive Performance Shaping Factors’ and this theme was reported in almost all literature included in this scoping review. This principally aims to reduce the risks in the work environment considering workflows, work environment, physical design including layout of medication storage, aswell as human factors [15]. Physical separation of LA medications through the use of technology such as Automated Dispensing Cabinets (ADC’s), making medications only accessible through locked and lidded single compartments and are examples of a constraint, barrier and forcing functions (refer to Supplementary Information: Table 1). Label design, inhouse pharmacy labelling and the use of technology, such as barcode scanning of medications at pharmacy distribution and administration were additional subthemes identified [8, 21, 23, 24, 29, 34, 37]. Estock et al. [21] demonstrated through a controlled simulation study under a high stress clinical situation that a redesigned medication label aligned to key medication safety recommendations improved the correct selection. Barcode scanning technology usage to ensure the correct selection of medication, independent of human factors was also suggested as a technology based solution [8], Independent double checking of medication labelling and packaging at the point of administration was suggested [8, 34], however the challenges with clinician acceptance may be a potential barrier.

This review highlighted that further research involving both quantitative and qualitative methodologies, such as surveys and semi-structured interviews, in addition to observational studies, may be useful in determining the effectiveness of interventions and the reduction in patient harm in the perioperative setting.

## Conclusion

Our review highlighted that LA incidents related to labelling and packaging of the primary container have been reported in the perioperative setting, resulting in patient harm. Risk reduction interventions have emerged that are not dependent on expensive, technology-based solutions providing an opportunity for organisations which is not cost prohibitive to translate these solutions into clinical practice. Healthcare facilities could use multiple LA interventions to guide quality improvement activities, within both the perioperative and pharmacy department settings. However, further research with robust methodologies are required to demonstrate the effectiveness of these interventions in preventing patient harm.

### Supplementary Information

Below is the link to the electronic supplementary material.

Supplementary file1 (DOCX 21 KB)
