# Supplementary material for: Look-alike medications in the perioperative setting: scoping review of medication incidents and risk reduction interventions
Source: Int J Clin Pharm. 2023 Sep 9;46(1):26–39. doi: 10.1007/s11096-023-01629-2 (PMC10830657; doi:10.1007/s11096-023-01629-2)
Supplement: Supplementary file 1 — Supplementary file1 (DOCX 21 KB) [file 11096_2023_1629_MOESM1_ESM.docx]

**Supplementary Information (SI)**

**Look-Alike Medications in the Perioperative Setting: Scoping review of medication incidents and risk reduction interventions**

**A.N. Ryan^1,2^ · K.L. Robertson^1,2^· B.D. Glass^2^**

Alexandra N Ryan 0000-0002-3098-1036

Kelvin L Robertson 0000-0001-8411-1864

Beverley D Glass 0000-0002-5444-3312

^1^. Pharmacy Department, Townsville University Hospital, 100 Angus Smith Drive, Douglas QLD 4810, Australia

^2^. College of Medicine & Dentistry, James Cook University, Townsville, Australia

Corresponding Author: Alexandra Ryan [Alex.Ryan@my.jcu.edu.au](mailto:Alex.Ryan@my.jcu.edu.au)

**Table 1**. Exerts of key definitions from the Australian Commission Safety and Quality in Healthcare (ACSQHC) Principles of the safe storage and selections of medications [15].

| **Term** | **Definition** |
| --- | --- |
| **Positive Performance Shaping Factors** | Provide a work environment that reduces the risks associated with the selection and storage of medicines. The physical design, layout and choice of medicine storage equipment and technology is conducive to safe selection, storage and preparation of LASA medicines.  An aspect of humans’ individual characteristics, environment, task or organisation that specifically improves human performance, thus reducing the likelihood of error (for example, task complexity; workflow; workload; time availability/urgency; process design; experience; training; fatigue; stress; culture). For instance, limit distractions in the environment when carrying out complex tasks. |
| **Standardisation** | Reduce risk of medicine selection error through standardisation of processes, systems or technology. Source commercially available products in the most ready-to-use formulation. |
| **Constraints, Barriers and Forcing Functions** | Physically separate look-alike medicines that present risks associated with selection and storage. Employ automated storage technology in pharmacy, or clinical/ward storage locations. If not automated, physically separate different brands and strengths of medicines by using shelf dividers or positioning on separate shelves.  Use constraints to restrict access to certain medicines or error-prone processes; require special education or conditions for prescribing, dispensing or administration of a medicine; require special authorisation for participation in certain tasks.  Prevent something from happening until certain conditions are met, i.e., a barrier that allows correct performance only (forcing function) |
| **Limiting Access** | Apply and communicate formulary restrictions related to medicines. Restrict stock (imprest) of certain concentrations, strengths or formulations in clinical or ward storage locations |
| **Differentiating items** | Alter the appearance of packaging and/or labelling to emphasise the difference between look-alike medicines. Alter the appearance of LASA medicine names on shelving and stock containers. |
| **Redundancy** | Implement practices (manual) or design features or warnings (electronic) to detect and prevent medicine selection errors. Employ independent second check (manual) for high risk or LASA medicines, or high-risk techniques involving medicines.  Implementing multiple pathways so that if the first pathway fails, a second pathway may detect the error and be successful, for instance, use of an independent double-check before administration of an intravenous dose of a medicine; barcode scanning during the dispensing or administration process. |

**Table 2.** Search strategy for electronic databases.

**Search key words/synonyms:**

anesthesia or anaesthesia or anesthetic* or anaesthetic* or "perioperative care" or "anaesthetic agent" or "anesthetic agent"

AND

"adverse drug event" or "drug error* or "medication error*" or "medication safety" or "risk management" or "patient safety" or "drug safety*" or "low technology safety" or " low-technology safety" or violation or "treatment error*" or "risk reduction or “drug labelling” or “drug packaging” or “drug stability” or “drug storage” or “product evaluation” or “look alike sound alike” or “look alike* sound alike*” or lasa

AND

“anaesthesia department hospital” or hospital* or hospitals or “operating rooms” or “operating theatre*” or “operating theatre*” or pharmacy or “pharmacy service” or pharmacist* or “clinical pharmacist*”

**Table 3.** Common factors associated with labelling and packaging concerns as per reported incidents from the Institute for Safe Medication Practices (ISMP) National Medication Errors Reporting Program (ISMP MERP) [39].

| **Aspect of Product Labelling/Packaging** | **Examples of Error-Prone Conditions** |
| --- | --- |
| Readability | - Clear or embossed labels without enough contrast to read the text - Small size text/font, label clutter, and poor legibility of labels - Manufacturer information competes in size and prominence with essential drug information |
| Product Name | - Brand and/or generic names less prominent than the graphic design, corporate dress, or company logos - Brand name extensions in which the brand name is already in use and well known for a totally different product/ingredient (see the Zantac reborn SAFETY brief starting in the bottom right column) |
| Dose, Strength, and/or Amount in Container | - Strength of injectables only expressed as a per mL concentration (e.g., mg per mL) rather than the total amount of drug per container volume - Dose/strength (or name) not visible on blister packs once separated - Different strengths of the same product not clearly differentiated - Dose, strength, and/or quantity expressed without a leading zero or with a trailing zero (e.g., .2mg [incorrect way] instead of 0.2mg [correct way]; 2.0mg [incorrect way] instead of 2mg [correct way]) - No space between the dose/strength and unit of measure (e.g., 10mg [incorrect way] instead of 10 mg [correct way]) - Commas not properly placed within numbers for large doses and strengths (e.g., 10000 [incorrect way] instead of 10,000 [correct way]) |
| Route | - Insufficient prominence given to the route of administration |
| Diluent | - Product name more prominent than ‘Diluent’ on the diluent container |
| Preparation | - Unclear admixture and/or product preparation instructions - Expiration date and storage instructions after reconstitution are absent |
| Warning/Cautionary Statements | - Absent warning/cautionary statements about proper drug use, such as: - Neuromuscular blocking agents: Warning, Paralyzing Agent - Insulin Pens: Single Patient Use - Drugs Requiring Dilution: Must Dilute Before Use - Transdermal Patches: Must Remove Before MRI Procedure - Using negative (instead of affirmative) language for warning statements (e.g., Not for Intrathecal Use [incorrect way] instead of For Intravenous Use Only [correct way]) |
| Differentiation | - Container labels look similar to another product from the same or different manufacturer - Lack of differentiation between products that have similar names - Poor use or the absence of colour to differentiate products - Transdermal patches are not easily identifiable on the skin |
| Expiration Dates/Lot Numbers | - Confusing expiration dates that do not follow the standard format of YYYY-MM-DD or YYYY-MM (or MMM if displaying the month in letters) - Expiration dates and lot numbers mistaken for each other when near each other and have a similar number sequence |
| Dosing Devices | - Dosing device not capable of measuring the recommended dose or with confusing measurement graduations - Oral dosing syringes with Luer connectors - Dosing devices that do not use only metric measurements |
| Barcode | - Linear barcode not present or unscannable on the immediate container, blister packaging, overwrap (intravenous [IV] bags) - Multiple barcodes on the label, only one of which can be used to verify the product - Barcode located over perforation in packaging - Barcode presented in a horizontal position and curved around the vial (unscannable) |
| Abbreviations and Symbols | - Error-prone abbreviations (e.g., U for units) or symbols used on the product label |
